# Gas conditioning during helmet noninvasive ventilation: effect on comfort, gas exchange, inspiratory effort, transpulmonary pressure and patient–ventilator interaction

**DOI:** 10.1186/s13613-021-00972-9

**Published:** 2021-12-24

**Authors:** Filippo Bongiovanni, Domenico Luca Grieco, Gian Marco Anzellotti, Luca Salvatore Menga, Teresa Michi, Melania Cesarano, Valeria Raggi, Cecilia De Bartolomeo, Benedetta Mura, Giovanna Mercurio, Sonia D’Arrigo, Giuseppe Bello, Riccardo Maviglia, Mariano Alberto Pennisi, Massimo Antonelli

**Affiliations:** 1 Department of Anesthesiology and Intensive Care Medicine, Catholic University of The Sacred Heart, Rome, Italy; 2 Anesthesia, Emergency and Intensive Care Medicine, Fondazione Policlinico Universitario A. Gemelli IRCCS, L.Go F. Vito, 00168 Rome, Italy

**Keywords:** Noninvasive ventilation, Respiratory Insufficiency, Humidity, Temperature

## Abstract

**Background:**

There is growing interest towards the use of helmet noninvasive ventilation (NIV) for the management of acute hypoxemic respiratory failure. Gas conditioning through heat and moisture exchangers (HME) or heated humidifiers (HHs) is needed during facemask NIV to provide a minimum level of humidity in the inspired gas (15 mg H_2_O/L). The optimal gas conditioning strategy during helmet NIV remains to be established.

**Methods:**

Twenty patients with acute hypoxemic respiratory failure (PaO_2_/FiO_2_ < 300 mmHg) underwent consecutive 1-h periods of helmet NIV (PEEP 12 cmH_2_O, pressure support 12 cmH_2_O) with four humidification settings, applied in a random order: double-tube circuit with HHs and temperature set at 34 °C (HH34) and 37 °C (HH37); Y-piece circuit with HME; double-tube circuit with no humidification (NoH). Temperature and humidity of inhaled gas were measured through a capacitive hygrometer. Arterial blood gases, discomfort and dyspnea through visual analog scales (VAS), esophageal pressure swings (Δ*P*_ES_) and simplified pressure–time product (PTP_ES_), dynamic transpulmonary driving pressure (Δ*P*_L_) and asynchrony index were measured in each step.

**Results:**

Median [IqR] absolute humidity, temperature and VAS discomfort were significantly lower during NoH vs. HME, HH34 and HH37: absolute humidity (mgH_2_O/L) 16 [12–19] vs. 28 [23–31] vs. 28 [24–31] vs. 33 [29–38], *p* < 0.001; temperature (°C) 29 [28–30] vs. 30 [29–31] vs. 31 [29–32] vs 32. [31–33], *p* < 0.001; VAS discomfort 4 [2–6] vs. 6 [2–7] vs. 7 [4–8] vs. 8 [4–10], *p* = 0.03. VAS discomfort increased with higher absolute humidity (*p* < 0.01) and temperature (*p* = 0.007). Higher VAS discomfort was associated with increased VAS dyspnea (*p* = 0.001). Arterial blood gases, respiratory rate, ΔP_ES_, PTP_ES_ and ΔP_L_ were similar in all conditions. Overall asynchrony index was similar in all steps, but autotriggering rate was lower during NoH and HME (*p* = 0.03).

**Conclusions:**

During 1-h sessions of helmet NIV in patients with hypoxemic respiratory failure, a double-tube circuit with no humidification allowed adequate conditioning of inspired gas, optimized comfort and improved patient–ventilator interaction. Use of HHs or HME in this setting resulted in increased discomfort due to excessive heat and humidity in the interface, which was associated with more intense dyspnea.

*Trail Registration* Registered on clinicaltrials.gov (NCT02875379) on August 23rd, 2016.

## Introduction

The ongoing COVID-19 pandemic dramatically highlighted the role of noninvasive management of acute hypoxemic respiratory failure [1–3]. In hypoxemic respiratory failure, there is raising interest on the possible benefit of helmet noninvasive ventilation (NIV), since a growing body of evidence suggests physiological and clinical advantages over both facemask and high-flow nasal oxygen [4–8]. Putative benefits of this technique include the possibility of delivering long-term treatments with high levels of positive end-expiratory pressure (PEEP) [9–11], which improve hypoxemia, mitigate self-inflicted lung injury and may improve clinical outcome [12–14].

For helmet NIV, gas-compressed mechanical ventilators are necessary to provide the high inspiratory flows needed to wash out exhaled CO_2_ from the high device-related dead space [15]. When a compressed-gas ventilator is used, dry and cold air is delivered to the patient, while a minimum level of absolute humidity of 15 mgH_2_O/L has been shown to be necessary to avoid patients’ discomfort and airway dryness during NIV [16]. Comfort and tolerability are key determinants of NIV success [17, 18]. Thus, gas conditioning is recommended during facemask NIV with compressed-gas ventilators [19–28], either by heated humidifiers (HH) or heat and moisture exchangers (HME), whose effects appear similar [16, 26–29]. These results may not be applicable to helmet NIV, as the interface is a 18-L mixing chamber that allows some degree of gas heating and humidification by the patient himself; the few available data during helmet continuous positive airway pressure (CPAP) indicate that artificial conditioning of the inspired gas may be necessary only as the flow washing the system exceeds 30–35 L/min [30, 31]. To our knowledge, no data ever assessed the effects during helmet pressure-support ventilation of the different strategies available for gas conditioning.

We conducted a randomized cross-over study to assess the effects of four different gas conditioning strategies during helmet pressure-support ventilation in hypoxemic patients, in terms of comfort, work of breathing, respiratory mechanics, patient–ventilator interaction and gas exchange.

## Methods

This prospective, physiological, randomized, cross-over trial was conducted in the 20-bed general intensive care unit (ICU) of a university hospital in Italy between February 2017 and January 2019. Data analysis and manuscript drafting were delayed due to the COVID-19 pandemic. The study protocol was approved by local ethics committee and prospectively registered on clinicaltrials.gov (NCT02875379). All enrolled patients provided written informed consent to participating in the trial and data analysis.

### Patients

Non-hypercapnic adult patients deemed by the attending physician to require respiratory support due to acute hypoxemic respiratory failure (PaO_2_/FiO_2_ ratio < 300) were screened for the enrollment. Exclusion criteria were: exacerbation of asthma or chronic obstructive pulmonary disease; clinical evidence of acute cardiogenic pulmonary edema; hemodynamic instability (systolic blood pressure < 90 mmHg or mean arterial pressure < 65 mmHg) and/or shock; metabolic acidosis (pH < 7.30 with normo- or hypocapnia); hypercapnia (PaCO_2_ > 45 mmHg); Glasgow coma scale < 13; recent gastric or abdominal surgery; non-collaborative patient.

### Interventions

**Fig. 1:**
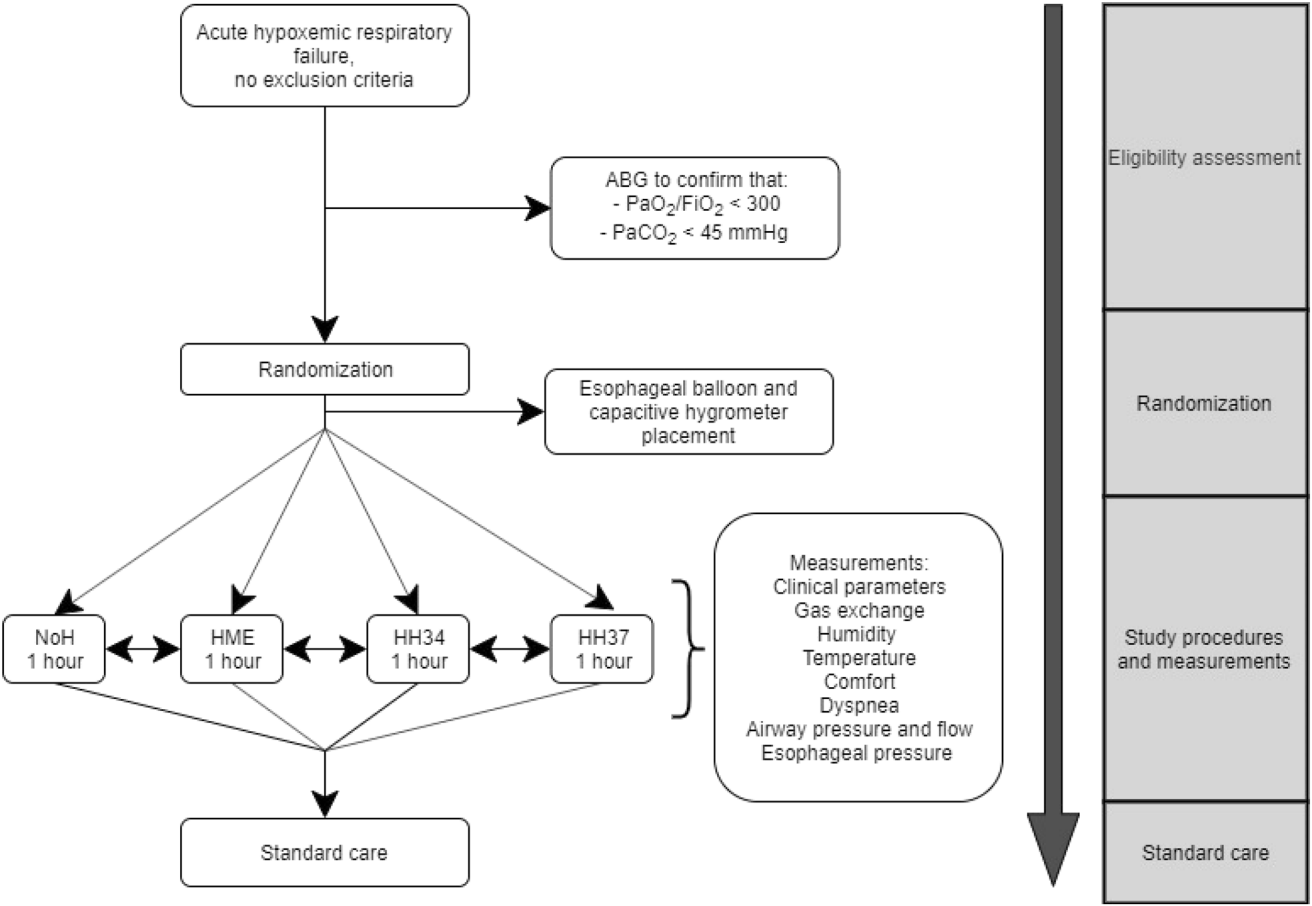
Study flowchart

Study flow diagram is shown in Fig. 1.

Patients underwent helmet NIV through a dedicated interface (Dimar, Mirandola, Italy), whose size was chosen according to neck circumference [9]. Patients were connected to a pneumatic mechanical ventilator (Drager Evita XL or Evita Infinity, Lubeck, Germany) [32]. The ventilator was set in pressure-support mode with the following settings: initial pressure support 10–12 cmH_2_O and then adjusted to attain a peak inspiratory flow of 100 L/min, up to a maximum of 20 cmH_2_O; PEEP 10–12 cmH2O; flow trigger 2 L/min and eventually increased in the presence of significant autotriggering; fastest pressurization time; expiratory trigger 30% of maximum inspiratory flow and eventually diminished in the case of double-triggering; maximum inspiratory time 1.0–1.2 s. FiO_2_ was titrated to obtain as SpO_2_ ≥ 92% and ≤ 98%. These settings were kept unchanged during the study steps.

**Fig. 2:**
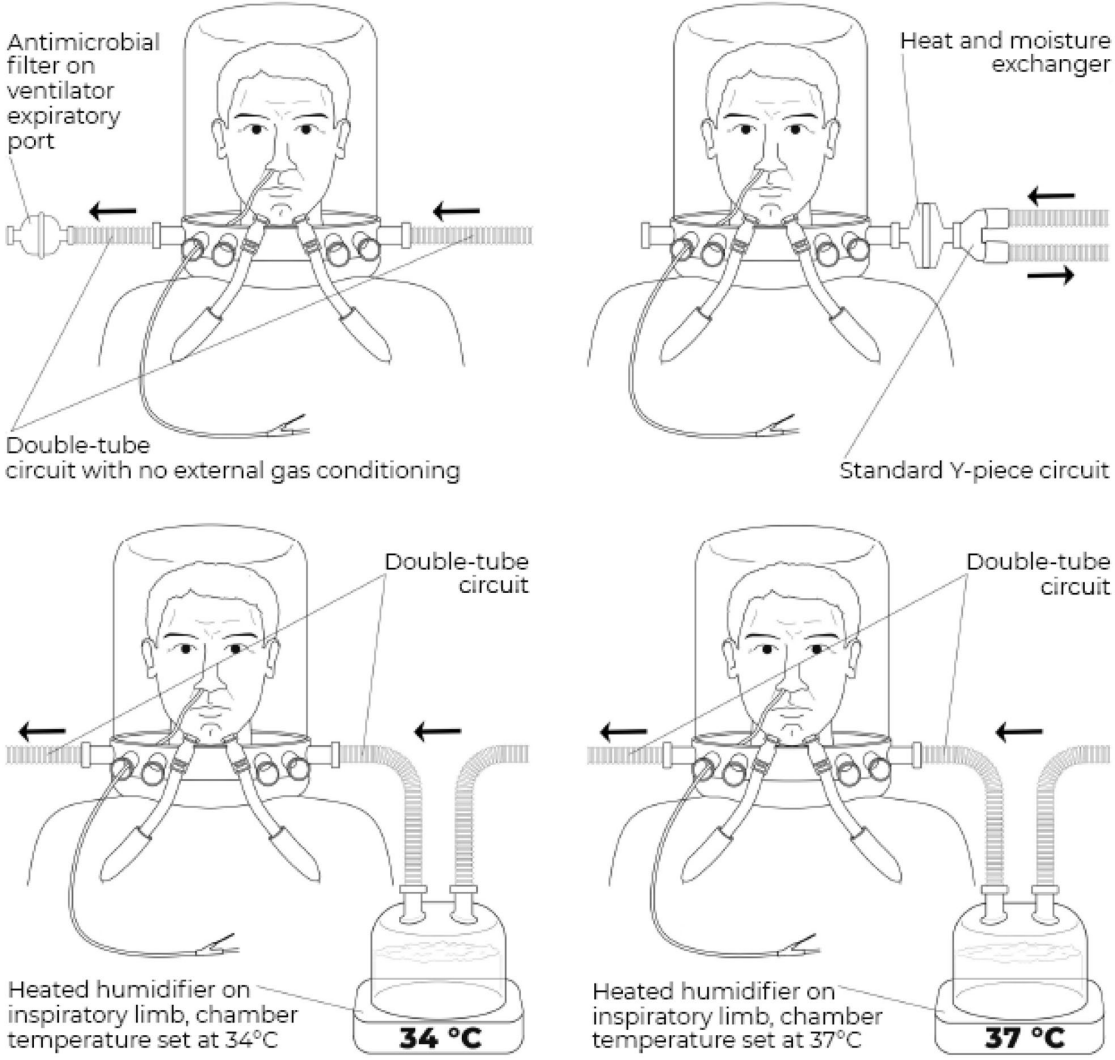
Study set-up. The four study steps are illustrated. Black arrows indicate the direction of flow (i.e., arrows pointing towards the patient indicate the inspiratory limb, whereas arrows pointing away from the patient indicate the expiratory limb). Polyfunctional nasogastric tube for the measurement of esophageal pressure is also illustrated. Upper left panel: NoH step (double-tube circuit with no external humidification). Upper right panel: HME step (standard Y-piece circuit with a heat and moisture exchanger). Lower left panel: HH34 step (double-tube circuit with a heated humidifier, with humidification chamber set at 34 °C on the inspiratory limb). Lower right panel: HH37 step (double-tube circuit with a heated humidifier, with humidification chamber set at 37 °C on the inspiratory limb)

Each patient received four gas conditioning strategies (*study steps*) in a random order, for a duration of 1 h each (randomization was performed with SAS random allocation software and enrolled patients were assigned to the allocated sequence through sealed opaque envelopes) (Fig. 2):

- Heated humidification (MR860, Fisher and Paykel healthcare, New Zealand) with humidification chamber set at 34 °C—double-tube circuit (*HH34* step);
- Heated humidification (MR860, Fisher and Paykel healthcare, New Zealand) with humidification chamber set at 37 °C—double-tube circuit (*HH37* step);
- Passive humidification with a heat and moisture exchanger (DAR™, Covidien, Medtronic, USA)—standard circuit with Y-piece (*HME* step);
- No external humidification provided—double-tube circuit (*NoH* step).

To avoid any carry-on effect, between each step, patients received a 15-min washout period on oxygen therapy.

After enrollment, a polyfunctional nasogastric tube provided with an esophageal balloon (Nutrivent, Sidam, Italy) was placed to measure esophageal pressure (P_ES_). Because of the impossibility to perform Baydur maneuver in nonintubated patient [33–35], the esophageal balloon was filled with 4 ml of air, which has been shown to be a non-stress volume providing reliability in a wide pressure range for this specific balloon [36]. To ensure intra-individual reproducibility, esophageal balloon was deflated and, after checking adequate zeroing, re-inflated before all measurements.

A capacitive hygrometer (Dimar, Mirandola, Italy) for measurement of absolute and relative humidity (AH and RH) and temperature was positioned inside the helmet through a dedicated port. The device was calibrated before all measurements.

### Measurements

Patient’s demographic and clinical characteristics and baseline room temperature and humidity were collected at study entry. During the study, patients received standard ICU monitoring (5-lead electrocardiogram, invasive blood pressure and SpO_2_).

At the end of each study step, clinical parameters (heart rate, arterial blood pressure, SpO_2_,) and arterial blood gases were collected, and patients were asked to rate the degree of dyspnea and device-related discomfort according to visual analog scales (VAS) for critically ill patients [5]. The examiner subjectively visually evaluated the presence and degree of condensation inside the helmet (*“fog effect”*) according to a 1-to-3 observation scale.

At the end of each step, after a stable breathing pattern was established and after ensuring stability of humidity and temperature measurements, a pressure transducer measured (sampling rate = 200 Hz) *P*_ES_ and a Fleisch-type pneumotachograph (n.2, Metabo, Lausanne, Switzerland) measured inspiratory flow and airway pressure (Paw). All signals were amplified, low-pass filtered, digitalized and transmitted to a personal computer with dedicated softwares for waveforms analysis (ICU Lab, Kleistek, Bari, Italy) and temperature and humidity data analysis (Humidity Dimar, E + E Elektronik Ges.m.b.H, Austria). All data from this recording were analyzed and results were averaged for each study step.

*P*_ES_, Paw and flow tracings where then reviewed offline and the following parameters were computed for each step:

- Respiratory mechanics and work of breathing indices: patient’s respiratory rate (number of negative deflections of *P*_ES_ per minute); ventilator respiratory rate (number of ventilator-delivered breaths per minute); inspiratory effort (Δ*P*_ES_, the negative inspiratory swing of *P*_ES_); simplified *P*_ES_ pressure–time product per breath (PTP_ES_: area under the curve of esophageal pressure during inspiration, chest wall recoil pressure was neglected due to the impossibility of performing occlusions, *P*_ES_) and per minute; dynamic transpulmonary driving pressure (Δ*P*_L_, the positive tidal swing in transpulmonary pressure, calculated as the difference between airway pressure and *P*_ES_) [5, 34, 35, 37]. Δ*P*_L_, albeit primarily influenced by tidal volume, also includes a resistive component due to airflow; we assumed that resistive pressure did not change significantly between the four study steps, thus allowing to take Δ*P*_L_ as a surrogate for transpulmonary driving pressure.
- Patient–ventilator synchrony: the following asynchronies were diagnosed by in-phase reading of esophageal and airway tracings [35, 38, 39], according to existing definitions and diagnostic criteria [39–41]:
  - Ineffective efforts: negative flexion of esophageal pressure (i.e., active inspiratory effort) without a corresponding delivery of a mechanical breath by the ventilator.
  - Autotriggering: delivery of a mechanical breath by the ventilator without a corresponding negative flexion of esophageal pressure.
  - Double cycling: delivery of two mechanical breaths by the ventilator corresponding to a single continuing negative flexion of esophageal pressure.
  - Premature expiratory cycling: termination of a mechanical breath delivered by the ventilator (as assessed by the flow tracing) preceding the end of the corresponding patient’s active inspiratory effort as assessed by the esophageal pressure tracing.

The overall burden of asynchronies was assessed by the asynchrony index, defined as the total number of asynchronies divided by the total respiratory rate (computed as the sum of the number of ventilator-delivered breaths, triggered or not, and ineffective efforts). Inspiratory and expiratory trigger delay asynchronies were not considered, since helmet NIV is known to be characterized by significant physiological trigger delays due to the compliance of the interface and its associated slow internal pressure buildup and decay [5, 11, 42].

For all calculations, start of inspiration was determined at the instant of *P*_ES_ initial decay, and end of inspiration was determined at the point of the respiratory cycle where 25% of time elapsed from *P*_ES_ maximal negative deflection towards its return to baseline [43].

### Endpoints

Primary endpoints were VAS discomfort, work of breathing indices and patient–ventilator synchrony. Main secondary endpoints were: humidity and temperature inside the interface, degree of *fog effect*, VAS dyspnea, respiratory rate, expired helmet minute volume, dynamic transpulmonary driving pressure.

### Sample size calculation

Given the physiological design of the study, we did not perform a formal sample size calculation. Consistent with previous investigations on the topic with similar endpoints [16, 30, 31], we planned to enroll 20 patients.

### Statistical analysis

Qualitative data are expressed as number of events (%) and continuous data as median [interquartile range]. Ordinal qualitative variables and non-normal quantitative variables distributions in the four study step were compared with the Wilcoxon/Kruskal–Wallis sum of rank test. Paired comparisons between steps were performed with the Wilcoxon test. All analysis was performed applying a bilateral hypothesis and results with *p* ≤ 0.05 were considered significant. Statistical analysis was performed with JMP® Pro, Version 13 (SAS Institute Inc., Cary, NC, USA). Manuscript figures were prepared with GraphPad Prism (GraphPad Software, La Jolla, CA USA).

## Results

Twenty patients were included. Patients’ demographic and baseline most relevant clinical features are shown in Table 1; main results of the study are displayed in Table 2.

**Table 1:** Demographics and baseline characteristics of enrolled patients

Age, years	72 [64–77]
Female sex , no. (%)	7 (35%)
Height, cm	168 [161–170]
Body mass index, kg/m^2^	28 [24–29]
Ideal body weight, kg	64 [57–66]
SAPS II	43 [34–51]
SOFA at study inclusion	4 [3–8]
Reason for ICU admission, no. (%)	
Medical	20 (100%)
Surgical	0 (0%)
Cause of respiratory failure, no. (%)	
Pulmonary, infectious	13 (65%)
Pulmonary, non-infectious	5 (25%)
Extrapulmonary	2 (10%)
Length of noninvasive respiratory support before enrollment, hours	0 [0–14]
Baseline respiratory support device at enrollment, no. (%)	
Venturi mask	1 (5%)
High-flow nasal oxygen	16 (80%)
Facemask noninvasive ventilation	1 (5%)
Helmet noninvasive ventilation	2 (10%)
Characteristics at enrollment	
PaO_2_/FiO_2_ ratio, mmHg	169 [112–279]
PaCO_2_, mmHg	33 [29–35]
pH	7.45 [7.41–7.50]
Heart rate, bpm	91 [81–109]
Mean arterial pressure, mmHg	93 [84–103]
Room temperature, °C	25 [24, 25]
Room absolute humidity, mgH_2_O/L	11 [9–12]
Room relative humidity, %	50 [41–52]
Sedation/analgesia during the study*, no. (%)	3 (15%)
Glasgow coma scale at study inclusion	15 [15–15]
Need for endotracheal intubation, no. (%)	9 (45%)
In-intensive care unit mortality, no. (%)	7 (35%)

Data are expressed as median [interquartile range], if not otherwise specified

^*^Two patients received remifentanil continuous infusion during the study, without dosage changes over the course of the study

**Table 2:** Main results of the study

	NoH	HME	HH34	HH37	p
Gas exchange					
PaO_2_/FiO_2_ ratio, mmHg	260 [216–320]	252 [207–317]	232 [188–303]	234 [201–292]	0.73
PaCO_2_, mmHg	30 [28–36]	34 [30–37]	31 [29–35]	34 [29–38]	0.46
pH	7.47 [7.42–7.49]	7.44 [7.41–7.48]	7.46 [7.42–7.49]	7.47 [7.41–7.49]	0.73
Gas conditioning, self-assessed symptoms and fog effect					
Absolute humidity, mgH_2_O/L	16 [12–19]^αβγ^	28 [23–31]^αε^	28 [24–31]^βζ^	33 [29–38]^γζ^	< *0.001*
Relative humidity, %	52 [40–63]^αβγ^	92 [81–97]^αε^	96 [83–100]^β^	100 [93–100]^γε^	< *0.001*
Helmet temperature, °C	29 [28–30]^α^	30 [29–31]^ε^	31 [29–32]^ζ^	32 [31–33]^αεζ^	< *0.001*
Dyspnea, VAS	2 [1–5]	3 [1–5]	4 [1–5]	4 [1–7]	0.46
Discomfort, VAS	4 [2–6]^γ^	6 [2–7]	7 [4–8]	8 [4–10]^γ^	*0.03*
Fog effect, 1–3 scale	0 [0–0]^αβγ^	1 [1–3]^αε^	2 [1–3]^βζ^	3 [3–3]^γζ^	< *0.001*
Respiratory mechanics and work of breathing indices					
Patient respiratory rate, bpm	26 [22–31]	26 [22–32]	27 [21–33]	26 [22–31]	0.99
Ventilator respiratory rate, bpm	31 [27–36]	31 [25–34]	32 [24–34]	30 [23–33]	0.43
Helmet minute ventilation, L*min^−1^	21 [20–27]	19 [16–24]^δε^	27 [23–30]^δ^	24 [20–32]^ε^	*0.009*
Δ*P*_ES_, cmH_2_O	7 [4–9]	9 [5–11]	6 [4–10]	7 [5–11]	0.54
Δ*P*_L_, cmH2O	21 [17–23]	17 [15–21]	20 [18–23]	21 [16–24]	0.21
PTP_ES_ per minute, cmH2O*sec*min^−1^	82 [40–127]	109 [32–172]	74 [34–137]	61 [39–112]	0.74
Patient–ventilator asynchronies					
Asynchrony index (AI), %	19 [4–51]	7 [2–33]	18 [5–35]	16 [4–40]	0.78
Ineffective efforts, %	1 [0–2]	1 [0–2]	1 [0–5]	2 [0–6]	0.55
Proportion of AI due to ineffective efforts, %	14 [0–71]	15 [0–63]	19 [0–64]	22 [0–61]	0.99
Double cycling, %	1 [0–5]^α^	0 [0–1]^αε^	0 [0–3]	1 [0–3]^ε^	*0.05*
Proportion of AI due to double cycling, %	7 [0–30]^α^	0 [0–7]^αε^	0.3 [0–12]	11 [0–26]^ε^	0.07
Premature expiratory cycling, %	7 [1–40]	2 [0–19]	6 [0–16]	4 [0–14]	0.72
Proportion of AI due to premature expiratory cycling, %	42 [9–80]	24 [0–74]	22 [6–56]	21 [13–56]	0.66
Autotriggering, %	0 [0–1]^βγ^	0 [0–2]^δ^	1 [0–8]^βδ^	1 [0–6]^γ^	*0.03*
Proportion of AI due to autotriggering, %	0 [0–2]^βγ^	2 [0–30]	14 [2–28]^β^	16 [3–39]^γ^	*0.01*
Hemodynamics					
Heart rate, bpm	83 [80–109]	90 [75–108]	90 [75–108]	90 [76–106]	0.98
Mean arterial pressure, mmHg	87 [73–104]	88 [78–106]	88 [72–101]	83 [74–103]	0.61

Data are expressed as median [interquartile range]

α indicates a p < 0.05 for paired comparisons between NoH and HME

β indicates a p < 0.05 for paired comparisons between NoH and HH34

γ indicates a p < 0.05 for paired comparisons between NoH and HH37

δ indicates a p < 0.05 for paired comparisons between HME and HH34

ε indicates a p < 0.05 for paired comparisons between HME and HH37

ζ indicates a p < 0.05 for paired comparisons between HH34 and HH37

### Gas exchange

Median [interquartile range] PaO_2_/FiO_2_ ratio at enrollment was 169 mmHg [112–279]. No differences in PaO_2_/FiO_2_, PaCO_2_, pH, heart rate and mean arterial pressure were detected between the study steps (Table 2).

### Gas conditioning

**Fig. 3:**
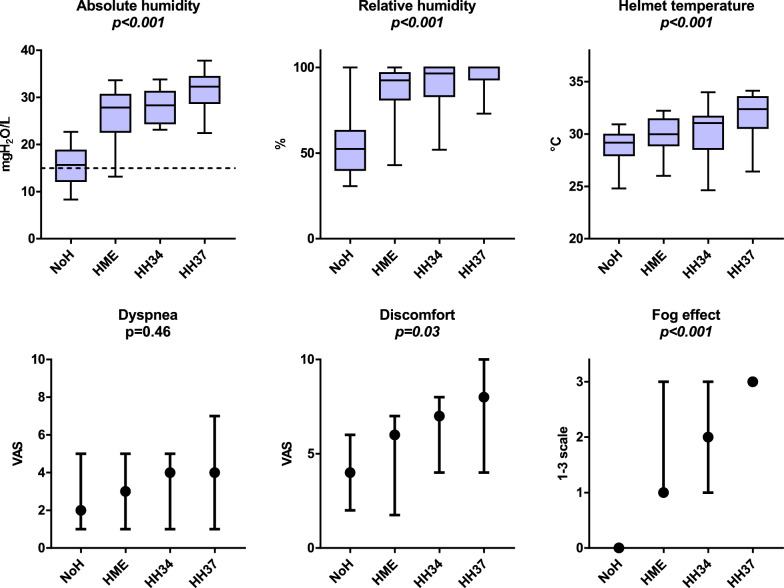
*Upper panels* show absolute humidity, relative humidity and temperature in the four study steps. Boxes indicate median [interquartile range] values, whiskers indicate minimum and maximum values. *Dotted line* in the absolute humidity panel marks the threshold of 15 mgH_2_O/L that has been indicated as the lowest humidity value to avoid discomfort during facemask NIV and CPAP [16]. When no external gas conditioning was applied (NoH step) both temperature and humidity were lower, albeit median absolute humidity was above 15 mgH_2_O/L, the 25^th^ percentile being 12 mgH_2_O/L (i.e., most patients received an adequate level of humidification). *Lower panels* show VAS dyspnea, VAS discomfort and subjective observator-assessed fog effect (on a scale from 1 to 3) in the four steps (black circles indicate median value, range indicates interquartile range). Discomfort and fog effect were lowest in the NoH step

These results are shown in Table 2 and Fig. 3. Absolute humidity, relative humidity and temperature inside the helmet interface were significantly lower when the NoH strategy was applied (all *p* < 0.001), displaying an increasing trend from the steps NoH to HME to HH34 to HH37. Median absolute humidity was above 15 mgH_2_O/L in all steps, notably being 16 [12–19] mgH_2_O/L in the NoH step.

### Self-assessed symptoms and fog effect

**Fig. 4:**
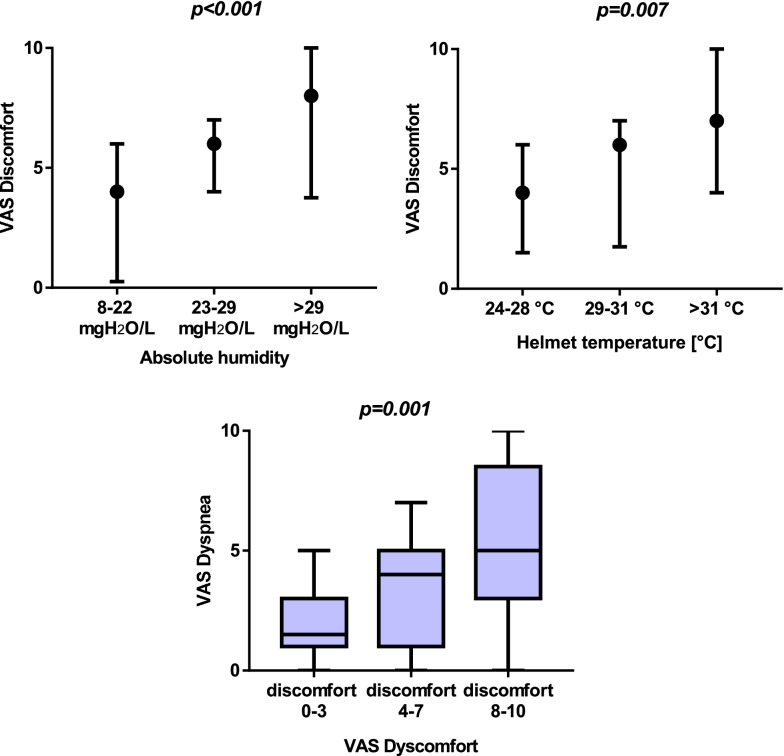
*Upper panels* show the relationship between absolute humidity (*left*) and temperature (*right*), respectively, and VAS discomfort. Values of humidity and temperature on the *x* axis are divided in lower, middle and upper tertiles in the whole cohort of measurements (i.e., all measurements from the four study steps). Medians (black circles) and interquartile ranges are displayed. Discomfort increased at raising humidity and temperature across all steps. *Lower panel* illustrates the relationship between VAS discomfort (presented on the *x* axis as low, intermediate and high discomfort levels) and VAS dyspnea in the whole cohort of measurements, showing how raising discomfort is associated to increasing subjective dyspnea (independently of the gas conditioning strategy used). Data in this panel are presented as a boxes-and-whiskers plot, the boxes indicating median [interquartile range] and the whiskers indicating minimum and maximum value

These results are shown in Table 2 and Fig. 3. VAS discomfort and observator-assessed fog effect were lower in the NoH step, increasing towards the higher-humidification steps (*p* = 0.03 and *p* < 0.001, respectively). VAS dyspnea was not significantly different between the study steps (*p* = 0.46). Across all study steps (all measurements taken into account), VAS discomfort increased at raising levels of absolute humidity (*p* < 0.001) and temperature (*p* = 0.007), as displayed in Fig. 4. Higher discomfort was associated to higher dyspnea (*p* = 0.001—*Fig. **4*).

### Respiratory mechanics and work of breathing

These results are displayed in Table 2. Neither patient nor ventilator respiratory rate were different between the study steps (*p* = 0.99 and *p* = 0.43, respectively). Total minute ventilation (i.e., the total system minute fresh gas flow) was slightly higher in HH34 and HH37 when compared to the non-actively humidified steps (*p* = 0.009). The analyzed work of breathing indices (Δ*P*_ES_, dynamic transpulmonary driving pressure and simplified esophageal PTP_ES_) along with Δ*P*_L_ did not differ between steps.

### Patient–ventilator interaction

As shown in Table 2, the asynchrony index was not significantly different between the four steps (*p* = 0.78), with a median asynchrony index of 16% [4–40%]. Overall, the most represented asynchrony was premature expiratory cycling, with an index of 4% when all measurements were taken into account [0–16%]. The rate of autotriggering, albeit low in all steps, was significantly higher in HH34 and HH37 steps (*p* = 0.03).

## Discussion

The main results of this randomized cross-over physiological study on gas conditioning during helmet pressure-support NIV can be summarized as follows:

1. Levels of humidity and temperature are significantly lower when no external gas conditioning is applied, when compared with the use of HME or HHs. Even with a NoH strategy, most patients are above the absolute humidity threshold of 15 mgH_2_O/L, which has been described as the minimum required humidification during NIV [16].
2. Subjective discomfort is lower when a double-tube circuit with no humidification is used, as it is the formation of fog effect inside the interface.
3. Overall, discomfort increased at raising levels of humidity and temperature, provided that the great majority of measurements above or around the abovementioned safety margin of 15 mgH_2_O/L. Increasing levels of discomfort appear to be related to worsening dyspnea in these patients.
4. The gas conditioning strategy does not appear to influence overall patient–ventilator synchrony, with the exception of an increased rate of autotriggering when HHs are used.
5. Respiratory rate, inspiratory effort, dynamic transpulmonary driving pressure and work of breathing are not affected by the gas conditioning strategy used. A slightly increased total helmet minute ventilation is seen when heated humidification is applied.

Two studies addressed the issue of gas conditioning during helmet continuous positive airway pressure (CPAP), and showed that humidification and comfort were optimal when no gas conditioning strategy was used during ventilator-delivered CPAP, while the application of a HH was needed at high continuous fresh gas flows (> 40 L min^−1^) with a PEEP valve [30, 31]. Notably, ventilator-delivered CPAP implies a lower fresh gas flow than continuous-flow CPAP, usually below the threshold of 30 L min^−1^, which is the minimum value of fresh gas flow suggested to avoid CO_2_ retention in the interface and significant rebreathing [15].

No previous data exist regarding the optimal gas conditioning strategy during helmet pressure-support ventilation, where system washout flow is equal or slightly superior to the system minute ventilation, typically lower than during high-flow CPAP but higher than during ventilator-delivered CPAP. This implies that existing data on helmet CPAP cannot be extrapolated to the setting of helmet pressure-support ventilation.

Our study demonstrates that, during helmet pressure-support ventilation, a double-tube circuit with no humidification allows adequate conditioning of the inspired gas, improves patient’s comfort and, when compared to active conditioning through heated humidifiers, may reduce ventilator autotriggering. Patient’s comfort appeared essential to prevent dyspnea. Both dyspnea and discomfort are important factors for successful noninvasive ventilation and avoiding the need for endotracheal intubation [17, 18, 44–47].

Helmet NIV is receiving growing attention and is being proposed as a first-line strategy in the noninvasive management of acute hypoxemic respiratory failure, as it may have physiological advantages and improve outcomes over facemask NIV and high-flow nasal oxygen [4–8]. Our study shines light on the rather unexplored but relevant issue of gas conditioning in the specific setting of helmet NIV and its effects on comfort and other patient-centered outcomes.

Our data indicate that an adequate minimal level of humidification is provided with all strategies during helmet NIV, including the absence of external gas conditioning. This is likely due to the relatively low total system washout flow (in all steps median helmet minute ventilation was below 30 L/min) when compared to high-flow CPAP, where usual flows range around 50 L/min: this allows the helmet interface to efficiently work as a mixing chamber where patient’s expired air directly contributes to the heating and humidification of ventilator-delivered dry inspired gas, without being massively washed-out as in high-flow CPAP [30]. Provided that minimal acceptable gas conditioning is guaranteed, our data indicate that excessive humidity and temperature may generate discomfort [31]: in our cohort *over*-humidification, rather than *under*-humidification, appeared to be related to patients’ discomfort. This explains the worsening discomfort when heated humidifiers are applied in this setting. Importantly, reduction of discomfort mediates concurrent reductions in the dyspnea score.

The gas conditioning strategy does not appear to influence gas exchange, work of breathing and overall patient–ventilator interaction (as measured by the asynchrony index). When individual asynchronies were analyzed, a small increase in the autotriggering rate with heated humidifiers was apparent, likely due to increased water condensation in the inspiratory limb of the circuit [48].

Despite the physiological nature of the study, our results may have relevant clinical implications. During helmet pressure-support noninvasive ventilation in hypoxemic patients, a double-tube circuit with no external humidification applied may be an acceptable gas conditioning strategy, since it does not affect work of breathing and oxygenation and appears to improve patient’s comfort and patient–ventilator interaction.

Our study has limitations:

1. The duration of each study step was limited to 1 h, which may preclude generalizability of the results to patients subjected to longer NIV treatments. Specifically, such a limited time could be insufficient to accurately assess respiratory discomfort directly related to higher airway dryness in case of under-humidification. However, study measurements were taken at the end of the 60-min periods after ensuring stability of physiological data, and the randomized cross-over design with 60-min consecutive periods of the present investigation is consistent with previous studies comparing different gas conditioning systems during CPAP or NIV with similar physiological endpoints [16, 28–30, 49]. We believe that this makes the results of our manuscript reproducible for longer-term treatments.
2. The small sample size may have precluded the possibility to draw specific conclusions on secondary outcome measures and particularly subgroup analyses. Nevertheless, similar sample sizes have been selected for physiological studies focusing on similar outcomes, and results on the primary outcomes show a strong statistical significance.
3. PaO_2_/FiO_2_ ratio, albeit below 300 mmHg, varies significantly between patients, with a relevant proportion of patients exhibiting mild hypoxemia (PaO_2_/FiO_2_ between 200 and 300). This may have diluted the sample of moderate-to-severe patients, which are expected to display higher inspiratory effort and total minute ventilation: it is possible that a sample of more severe patients with higher helmet minute ventilation could reach higher median washout flows, possibly exceeding 30–40 L/min and thus physiologically resembling the abovementioned condition of high-flow CPAP [30]. In such a setting, our conclusions may not be applicable, as the risk of under-humidification could dictate the use of external gas conditioning strategies.

## Conclusion

During helmet pressure support NIV, a double-tube circuit with no external gas conditioning ensures adequate humidity and heat of the inspired gas, improves comfort and patient–ventilator interaction. Use of HHs or HME in this setting may generate excessive humidity and heat in the interface, finally increasing discomfort, which is associated to worse dyspnea.

## Data Availability

The datasets used and/or analyzed during the current study are available from the corresponding author on reasonable request.

## Abbreviations

ICU: Intensive care unit
NIV: Noninvasive ventilation
PEEP: Positive end-expiratory pressure
HH: Heated humidifier
NoH: No humidification
HME: Heat and moisture exchanger
ΔP_L_: Dynamic transpulmonary driving pressure
ΔP_ES_: Esophageal pressure inspiratory swing
PTP_ES_: Esophageal pressure inspiratory pressure–time product
